# Donor Intra-Center Absorption to Resonant States in Quantum Wells: Analysis of Peak Shapes

**DOI:** 10.3390/nano16110701

**Published:** 2026-06-05

**Authors:** Volodymyr Akimov, Viktor Tulupenko, Roman Demediuk, Anton Tiutiunnyk, Carlos A. Duque, Alvaro L. Morales, David Laroze, Miguel Eduardo Mora-Ramos, Igor Fodchuk, Tamara González-Vega

**Affiliations:** 1 Facultad de Ciencias Básicas, Universidad de Medellín, Medellín 050026, Colombia; 2 Grupo de Materia Condensada-UdeA, Instituto de Física, Facultad de Ciencias Exactas y Naturales, Universidad de Antioquia, Calle 70 No. 52-21, Medellín 050010, Colombia; 3 Physics Department, Donbas State Engineering Academy, 84300 Kramatorsk, Ukraine; 4 Mathematics, Physics and Computer Science Department, Donbas State Pedagogical University, 84116 Sloviansk, Ukraine; 5 Departamento de Física, Facultad de Ciencias, Universidad de Tarapacá, Arica Casilla 7D, Chile; 6 Instituto de Alta Investigación, Universidad de Tarapacá, Arica Casilla 7D, Chile; 7 Centro de Investigación en Ciencias-Instituto de Investigación en Ciencias Básicas y Aplicadas, Universidad Autónoma del Estado de Morelos, Cuernavaca CP 62209, Mexico; 8 Department of Urban Planning and Urbanism, Yuriy Fedkovych Chernivtsi National University, 2, Kotsiubynsky Str., 58012 Chernivtsi, Ukraine; 9 Facultad de Ciencias de la Vida, Universidad Viña del Mar, Agua Santa 7055, Rodelillo, Viña del Mar 2572007, Chile

**Keywords:** semiconductor heterostructures, point defects, resonant impurity states, intra-center absorption

## Abstract

The oscillator strengths of absorptive transitions from the ground to the resonant excited impurity states for the impurity positioned in and near the GaAs/AlGaAs rectangular quantum well are studied. Due to the resonant nature of the final states, the absorption peaks are broadened. The shape of the peaks is reproduced numerically as a function of impurity position with respect to the well and the well width. Peak parameters, such as maximum, broadening, and integral absorption, are analyzed numerically; the Fano parameter is considered qualitatively.

## 1. Introduction

In the contemporary landscape of solid-state physics and materials science, semiconductor materials of diverse dimensionalities and chemical compositions are recognized as the primary foundation for next-generation optoelectronic devices. By precisely engineering electronic band structures, quantum confinement effects, and lattice parameters, these material platforms can be tailored to span an extraordinary operational bandwidth, stretching from the terahertz [1,2,3] and far-infrared regimes to the deep ultraviolet spectrum [4]. Achieving such vast spectral coverage often relies on the transition from traditional bulk systems to low-dimensional architectures, such as 2D heterostructures [5,6,7,8], quantum wells and lead-free halide double perovskites [9]—which allow the precise manipulation of transition matrix elements and oscillator strengths. Consequently, these versatile material configurations are driving critical breakthroughs in technologies ranging from high-frequency wireless communications and infrared scene detection to solar energy harvesting and UV sterilization.

Point defects, including impurities in semiconductors and semiconductor nanostructures that provide localized electronic states, have recently attracted much attention as a promising source of single-photon generation and detection [10,11]. They are often referred to as “Color centers” [12] and may be important components of integrated photonics and quantum technologies, with many applications in sensing, quantum key distribution and quantum computing [13]. On the other hand, classical radiation generation based on continuous bands of electronic states has the advantage of sheer intensity and is indispensable in integrated photonic circuits, at least as a pump source for single-photon processes. As a result, the operation of an integrated photonic device is expected to involve both classical and non-classical generation/absorption. Moreover, understanding carrier transition mechanisms and resonance-related optical responses in semiconductor nanostructures is also relevant to a broader range of designs for integrated semiconductor functional devices, including photonic and energy-conversion systems [14]. Thus, the interaction between continuous and discrete electronic states and its influence on optical processes in semiconductor structures is of both fundamental and applied significance.

**Figure 1:**
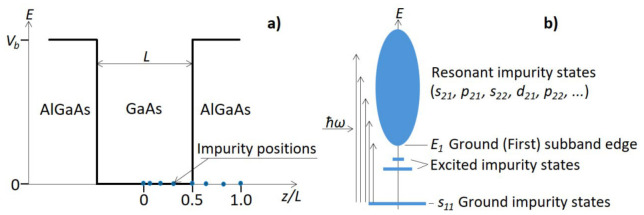
(**a**) Energy profile of the structures under study. (**b**) Optical transitions considered.

In this work, we investigate a structure that combines discrete and continuous electronic spectra, specifically a semiconductor quantum well doped within or in the vicinity of the well by a hydrogenic impurity. We consider the structure depicted in Figure 1a: a layered GaAs/AlGaAs nanostructure grown in the z-direction, with AlGaAs layers providing potential barriers and a GaAs layer providing a lower potential forming a rectangular quantum well (QW). One reason for selecting the GaAs/AlGaAs system for this study is to avoid band structure complications associated with intrinsic strain. In contrast to highly mismatched systems in which growth-induced strain triggers significant deformation [15,16], the lattice mismatch in this specific material couple is negligible [17], ensuring a strain-free potential profile. The motion of electrons inside the well is restricted in the z-direction, but they can move freely in the plane perpendicular to the z-axis, so the electronic states consist of a number of so-called space-quantized sub-bands (in addition to a 3D continuum of electronic states above the barriers). The impurity atoms in this region can be ionized, thereby distorting both the sub-bands and the 3D continuum. The result is a Rydberg-like series of (quasi) localized states under each sub-band (Figure 1b). If the well is sufficiently narrow and the distance between the sub-bands exceeds the impurity binding energy (we use this term to denote the energy distance between the lowest impurity state related to a sub-band and the edge of the corresponding sub-band), then impurity states appear inside the lower sub-band. Such quasi-localized states are called resonant states (by analogy with the well-known phenomenon of Fano resonance [18]). Mathematically solving the Schrödinger equation for a structure with the Coulomb impurity potential, we obtain both discrete localized impurity states for energies below the bottom of the ground sub-band and quasi-local ones within this sub-band, but genetically related to the second sub-band. Similar quasi-localized states are also found in other, higher-lying sub-bands. In most cases, the wave functions (WFs) of impurity states are similar to the WFs of the nearest higher sub-band, but at some energies, the solution has a significant localized part, so it can be used to calculate the characteristics of optical transitions between non-resonant (localized) and resonant (quasi-localized) states formed by the same impurity center, i.e., for intra-center transitions (Figure 1b).

Previously [19], we described in detail the numerical process for determining both the wave functions and energies of impurity states in quantum wells for both non-resonant and resonant states. In that work, we also proposed a new classification of these states based on the shape of the corresponding wave functions, since the conventional classification based on the equivalence of hydrogen-like impurity states in bulk semiconductors and the energy states of the hydrogen atom is not entirely suitable for characterizing impurity states in a quantum well, especially for resonant states. In our calculations, we used a modification of the expansion method, first proposed in [20], that exploits the azimuthal symmetry of the structure. Then [21], we presented wave functions for resonant and non-resonant impurity states and discussed their properties.

One of the main measurable characteristics of the semiconductor structures used for optoelectronic purposes is the absorption spectrum [22,23]. In this paper, we calculate the matrix elements and oscillator strengths for optical transitions between the impurity ground state ($s_{21}$ in our classification) and some of the first resonant states as functions of the transition energy, the quantum well width, and the impurity position relative to the well. Knowledge of the resonant wave functions allows us to calculate the absorption spectrum of such transitions at 0 K. As expected, the spectrum consists of several peaks. Our method allowed us to reproduce the exact shapes of these peaks, numerically fit them with a Lorentz–Cauchy distribution with a Fano resonance correction, and determine the corresponding parameters (peak broadening, peak height, energy position, and Fano parameter).

## 2. Assumptions and Limitations

Calculations were performed assuming the isotropic effective-mass approximation, and the well profile was treated as ideally rectangular. The impurity is treated as hydrogenic. The shapes of the spectral lines are calculated using the resonant nature of the final states, although the actual line broadening will also include contributions from other mechanisms. The reasons for choosing our structures and parameters are as follows. Material: GaAs/AlGaAs is a model material for analytical and numerical studies, as it has favorable conduction band characteristics for calculations, namely good sphericity and acceptable parabolicity, is free of internal stress, and has developed and accessible manufacturing technology. We expect qualitatively similar results for other materials. Quantum well width: For wells wider than 60 nm, the $s_{21}$ state (which is associated with the second sub-band) is below the first sub-band and is not resonant. In addition, the transition energies for such quantum wells are very small (in the THz range) and are no longer of interest to us. In wells smaller than 10 nm wide, there are not enough sub-bands to apply our expansion method with sufficient numerical precision. Impurity position: The impurity atom is inside and near the quantum well; if it is too far from the well, the corresponding wave functions become indistinguishable from the wave functions of the sub-bands.

## 3. Mathematical Model

### 3.1. Reference Frame

The model implies the axial symmetry of the problem with cylindrical coordinates *z*, *R*, and θ. The longitudinal *z*-axis passes through the impurity atom and is directed perpendicular to the QW layer, with *z* = 0 corresponding to the QW center. The polar axis *R* has an arbitrary direction parallel to the QW, and θ is the angular coordinate or azimuth.

### 3.2. Units

Here in formulas, we use the International System of Units (SI). In many cases, we present results in effective Rydberg as an energy unit 1 Ry = $\frac{\hbar^{2}}{2m^{*}r_{B}^{2}}$, where *ℏ* is the Planck constant, $m^{*}$ is the effective mass of the material, and $r_{B}$ is the effective Bohr radius $r_{B}$ = $\frac{4\pi \varepsilon_{0}\varepsilon \hbar^{2}}{m^{*}e^{2}}$, where *e* is the unit charge, $\varepsilon_{0}$ is the dielectric permittivity of vacuum, and ε is the dielectric permittivity of the material. The advantage of Rydberg as a unit of energy is its scalability, so our results can be easily compared with those obtained for other materials. We expect that our qualitative results may also be valid for the materials in which the approximation of effective mass is applicable; however, the electron dispersion is not parabolic, and the effective mass is not isotropic. For our material 1 Ry = 5.8meV, and for the remaining material parameters, please see [19]. For the *z*-dimension, we often use the units of quantum well width *L*, since we consider QWs with different widths. For *R*, we use nanometers.

Oscillator strengths for the transitions between states *p* and *q* can be calculated as:

(1) $$f_{pq}=\frac{2\;m^{*}}{3\;\hbar^{2}}\left(E_{p}-E_{q}\right)M_{pq}^{2}\;,$$

where $m^{*}$ is the effective mass, and $E_{i}$ is the energy of the *i*-th state. In case of transitions within the same band, $M_{pq}$ is a dipole matrix element

(2) $$M_{pq}=\left|\int_{V}\Psi_{p}^{*}\vec{r}\Psi_{q}\;dV\right|\;.$$

In our case of axial symmetry, cylindrical coordinates, and the fact that ψ(R,z) is a real number for both wavefunctions (WFs), this can be written as

(3) $$M_{pq}=2\pi \int_{z=-\infty }^{\infty }\int_{R=0}^{\infty }\psi_{p}\left(R,z\right)\;z\;\psi_{q}\left(R,z\right)\;R\;dR\;dz\;.$$

Here, we skip the matrix elements for dimensions other than *z*, because the dipole matrix element prohibits transitions between states of the same symmetry. Note that our mathematical model uses azimuthal symmetry about the *z*-axis, so the *R* components of the matrix elements (perpendicular to *z*) are always equal to 0. This means that our calculated oscillator strengths effectively describe the absorption of radiation polarized strictly along the structure’s growth direction.

Realistic experimental conditions often involve structural inhomogeneities—such as variations in interface roughness, impurity distributions, and other point defects—as well as additional symmetry-breaking effects that can influence the predicted absorption behavior. While these effects may contribute to the total oscillator strength by introducing R-components, they only affect R-polarized photons in terms of measurable absorption. During the experiment, these components can be easily removed using a polarization filter.

The WFs used in matrix elements should be normalized as follows:

(4) $$\int_{V}{\left|\Psi \right|}^{2}\;dV=2\pi \int_{z=-\infty }^{\infty }dz\int_{R=0}^{\infty }\psi {\left(R,z\right)}^{2}\;R\;dR=1\;.$$

### 3.3. Wave Functions

To calculate the impurity wave functions, we used the algorithm described in [19], based on the mathematical model first proposed in [20]. The problem is formulated as time-independent, and the WF is found by solving the Schrödinger equation:

(5) HΨ(R,θ,z)=EΨ(R,θ,z).

The Hamiltonian of the problem, written in SI, consists of the kinetic part, the QW potential *V*, and the Coulomb potential of the impurity atom:

(6) $$H=-\frac{\hbar^{2}}{2m^{*}}\frac{\partial^{2}}{\partial z^{2}}-\frac{\hbar^{2}}{2m^{*}}\left(\frac{\partial^{2}}{\partial R^{2}}+\frac{1}{R}\frac{\partial }{\partial R}+\frac{1}{R^{2}}\frac{\partial^{2}}{\partial \theta^{2}}\right)+V\left(z\right)-\frac{e^{2}}{4\pi \varepsilon_{0}\varepsilon \sqrt{R^{2}+{\left(z-z_{0}\right)}^{2}}}\;,$$

where $z_{0}$ is the impurity position on the *z* axis (impurity position on the *R* axis is always 0 in our reference system). $V\left(z\right)=\left\{\begin{array}{cc} V_{b}, & |z/L|>1/2\\ 0, & |z/L|<1/2 \end{array}\right.$ is an energy profile of a rectangular quantum well, *L* is the well width, and $V_{b}$ is the well barrier height, also known as the conduction band discontinuity.

The impurity WF is expressed as an expansion over the eigenvalues of QW $\xi_{j}\left(z\right)$, which correspond to a one-dimensional Hamiltonian similar to *H*, but without the Coulombic part and the kinetic term dependent on *R* and θ. Below $\xi_{j}\left(z\right)$ are the wave functions of the quantum well sub-bands. The numerical determination of $\xi_{j}\left(z\right)$ is a trivial problem, which we will not describe here. Using the advantage of the axial symmetry of the system, the impurity wave functions are presented as

(7) $$\Psi \left(R,\theta ,z\right)=e^{ia\theta }\psi \left(R,z\right)=e^{ia\theta }\sum_{j}f_{j}\left(R\right)\;\xi_{j}\left(z\right)\;,$$

with *i* being an imaginary unit, *a* as an azimuthal quantum number, and *j* denoting the quantum well sub-bands, which are expansion members. $f_{j}\left(R\right)$ is found numerically by solving a linear matrix equation

(8) Mf=λ,

where vector λ represents a boundary condition for the impurity WF far away from the impurity center, and *M* is some square matrix (please see in detail in [19] for how to calculate its elements). Here we only note that the matrix components depend on the energy *E*, the sub-band wave functions $\xi_{j}\left(z\right)$, the impurity position $z_{0}$, and the azimuthal quantum number *a*. f is a vector representing $f_{j}\left(R\right)$ for several considered expansion elements *j* and is discretized by uniform tabulation with respect to *R* from 0 to some $R_{\max}$ (again see [19] for details). $R_{\max}$ is a key calculation parameter, implying that the wave function to be sought is well localized within the $R=0\ldots R_{\max}$ interval. In this case, the calculation results should converge as $R_{\max}\to \infty$.

For impurity states located below the first sub-band of the QW, Equation (5) has solutions only at certain energies. These solutions form a series of Rydberg-type (non-resonant) states. Mathematically, in Equation (8), it is reflected by the vector λ = 0, and the equation can only be solved when the determinant of the matrix M is zero. At higher energies, the impurity state interacts with a continuum of the QW sub-bands. This interaction can be interpreted as a hybrid wave function that combines localized and sub-band WFs, resulting in an effect known as Fano resonance [18]. In general, such a hybrid WF exists at any energy above the bottom of the lower sub-band, but the influence of the impurity is significant only in the vicinity of some specific energies known as resonant energies. Far from these energies, the hybrid WF is reduced to a sub-band WF, which is the product of the WF confined in the *z* direction $\xi_{j}\left(z\right)$ and a plane wave corresponding to a free electron in the QW plane $\varphi \left(R\right)=e^{ikR}$. In our model, this is reflected in the requirement that the vector λ should represent a plane wave of a free electron φ(R) for the corresponding sub-bands. Using the axial symmetry and parabolicity, we can express *k* in terms of energy $k=\frac{1}{\hbar }\sqrt{2m^{*}E}$. Thus, we obtain the components of the vector λ as

(9) $$\lambda_{j}\left(R\right)=h\left(E_{j}-E\right)\;e^{i\;\frac{R}{\hbar }\sqrt{2m^{*}\left(E_{j}-E\right)}}\;,$$

where $E_{j}$ is the energy minimum of the *j*-th sub-band, *h* is the Heaviside function (which includes resonant boundary conditions for energies within the *j*-th sub-band). Now, in Equation (8), the vector λ is a discrete version of $\lambda_{j}\left(R\right)$, just as the vector f is a discrete version of $f_{j}\left(R\right)$. For the actual calculations, we simply used $\lambda_{j}\left(R\right)=h\left(E_{j}-E\right)$ instead of Equation (9), because our numerical experiments did not show any significant difference in the results compared to those obtained using Equation (9), and this simplified version gives us the advantage of having a real (R,z) factor of WF (Equation (7)), which is good for visual inspection of the results.

In addition, in this work, unlike [19,20,21], when calculating the resonant WFs, we used modified Bessel functions *K* and *I* instead of the usual *J* and N(Y), as should be the case according to [20]. Below, we provide some clarification on this change. Visually, the impurity WFs obtained using ordinary Bessel functions look like WFs obtained using modified Bessel functions superimposed on the real part of the plane wave of a free electron in a sub-band. This similarity is due to the fact that, for the case of WFs close to resonant energies (which are of interest to us), the contribution of plane waves is small, and the results obtained with the Bessel functions *J* and N(Y), and with the modified Bessel functions *K* and *I* are very similar. However, for energies far from resonant, the contribution of plane waves becomes more significant. To calculate the oscillator strengths using Equation (1), both wave functions must be normalized in 3D. Therefore, in our model, both states must be localized in 3D, but in some cases, this is difficult to achieve using a standard Bessel function. At the same time, proper normalization is crucial for the shape of the peaks since they are obtained by comparing oscillator strengths at different energies. Normalizing the hybrid wave function within $R_{\max}$ makes the result unstable with respect to $R_{\max}$, and in some situations, we were unable to achieve reliable convergence within numerically controlled $R_{\max}$ using conventional Bessel functions. On the other hand, in some situations, when using ordinary Bessel functions, the results converged and agreed well with the results obtained using modified Bessel functions. Even in situations of poor quantitative convergence, the qualitative results of the standard and modified cases were similar. Thus, all the presented results were obtained using modified Bessel functions. Physically, this can be interpreted as if we were studying only the addition of intra-center transitions to the oscillator strength and ignoring transitions from the center to the sub-band.

Unlike modified Bessel functions, standard Bessel functions exhibit oscillatory behavior and converge very slowly to zero. This oscillatory nature reflects the physical interaction between the resonant impurity WF and the plane-wave component of the sub-band WF. For numerical implementations using finite differences, these rapid oscillations necessitate an extremely fine grid step. Because the matrix dimension in Equation (8) scales with the fourth power of the number of steps in R, the step size becomes the primary limiting factor for our method’s computational precision.

Furthermore, the oscillations of standard Bessel functions exert a greater influence on the oscillator strength at larger values of R (i.e., far from the impurity atom). Consequently, substituting standard Bessel functions with modified ones implies that the resonant impurity wave functions constructed via this approximation—while acceptable for localized transitions between the ground and excited resonant WFs of the same impurity center, as confirmed by our comparative calculations—may prove inadequate for modeling photonic effects involving non-localized states (such as center-to-sub-band transitions), spatially separated states, or even transitions between distinct resonant states of the same center.

## 4. Classification of Impurity States

Here we use the classification of impurity states proposed in [19]. We use a combination of three quantum numbers *a*, *b*, *c* as subscripts for energies $E_{abc}$ and wave functions $\psi_{abc}\left(R,z\right)$. *a* is a quantum number explicitly presented in Equation (7) as an exponent. We denote it with the letters “*s*” (a = 0), “*p*” (a = 1) and “*d*” (a = 3) by loose analogy with the hydrogen atom orbitals. *b* and *c* are quantum numbers presented by digits starting from 1. *b* denotes the sub-band of the QW to which the impurity state is attached. It can be visually identified by the *z*-section of its WF: it qualitatively repeats the WF of the corresponding sub-band $\xi_{j}\left(z\right)$. On the other hand, *c* denotes a series of impurity states with fixed values of *a* and *b*, which form a series approaching the lower boundary of the corresponding sub-band to which the given impurity state is tied.

## 5. Calculating Spectra and Peak Shape Fitting

We calculated oscillator strengths of the transition from the ground impurity state $s_{11}$ to some states with energies between the first and second sub-bands, that is, to impurity states that are in resonance with the continuous energy spectrum of the first sub-band. It is obvious that the sum of such curves for all quantum numbers “*a*” is proportional to the intra-center absorption spectrum of our structures at a temperature of 0K in the one-electron approximation. The curves are expected to consist of a series of peaks; each peak corresponds to the energy of the $E_{abc}-E_{s11}$ transition. Since we are considering the resonance of impurity states with continuous states of the corresponding sub-band, one can expect that such peaks, instead of having the classical shape of a Lorentzian bell, will have an asymmetric Fano resonance shape. We identified and analyzed some peaks by fitting them to the Fano resonant formula [18]. This formula is

(10) $$f_{F}=f_{0}\;\frac{{\left(1+\frac{E-E_{0}}{q\gamma }\right)}^{2}}{1+{\left(\frac{E-E_{0}}{\gamma }\right)}^{2}}\;$$

where $E_{0}$ is the resonant energy, *q* is the Fano parameter, γ is the resonance broadening, and $f_{0}$ is the transition intensity. By fitting, we can obtain all four parameters. In the case of q→±∞, the formula reduces to the Lorentz–Cauchy distribution

(11) $$f_{L}=f_{0}\;\frac{1}{1+{\left(\frac{E-E_{0}}{\gamma }\right)}^{2}}\;.$$

In this case, the peak is symmetrical, $f_{0}$ is the peak maximum, found at $E=E_{0}$, and γ is the half-width at half-height of the peak. In the opposite case, when q=0, the curve is again symmetrical, but instead of a peak, it forms an absorption minimum with a similar bell-like shape. All other values of *q* indicate peak asymmetry of different grades. Note that in this case, the peak maximum differs from the resonance energy, and depending on other parameters, the difference can be significant. The same is true for $f_{0}$: in general, it differs from the peak maximum magnitude. Physically, the magnitude of the Fano parameter can be interpreted as the ratio of the resonant to the background scattering amplitude.

## 6. Results and Discussion

**Figure 2:**
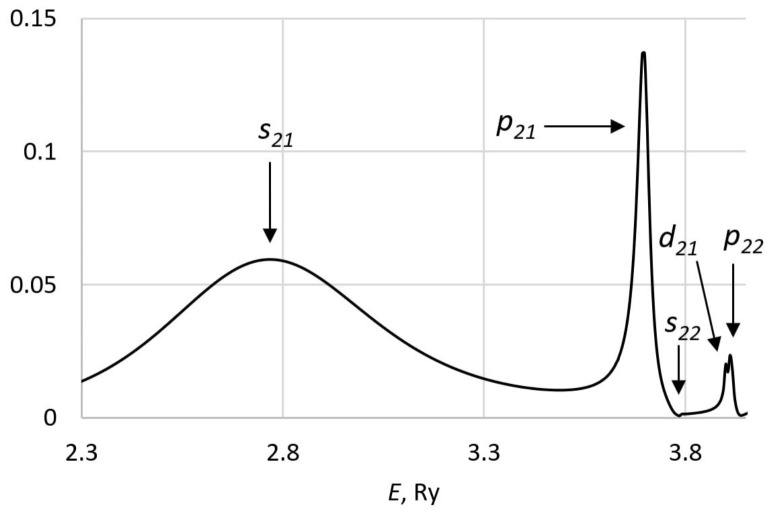
Oscillator strengths of transitions from the ground impurity state $s_{11}$ to the resonant impurity states attached to the second sub-band vs. transition energy. L=30 nm, z=0.2 L.

In Figure 2, we present the curve of calculated oscillator strengths for intra-center transitions from the ground impurity state $s_{11}$ to the states with energies between the first and second sub-bands, which are the lowest resonant impurity states. The curves for all the QW widths and impurity positions used have the same qualitative behavior, so we only use one of them as an example. It is worth mentioning that multiplying the oscillator strength by the difference in concentrations of the initial and final states and by some material-dependent constants allows us to obtain the absorption coefficient used in Beer’s law. Thus, the curve shown in Figure 2 represents the absorption spectrum of the structure in arbitrary units at *T* = 0 K when the impurity atoms are not ionized. Technically, this curve is obtained as the sum of the corresponding curves for the final “*s*” states (*a* = 0 in Equation (7)), the final “*p*” states (*a* = 1), and the final “*d*” states (*a* = 2). Transitions to the states with *a* > 2 are not considered here, since, as our calculations have shown, their amplitudes are significantly smaller, and their broadening is significantly greater than those presented in Figure 2.

**Figure 3:**
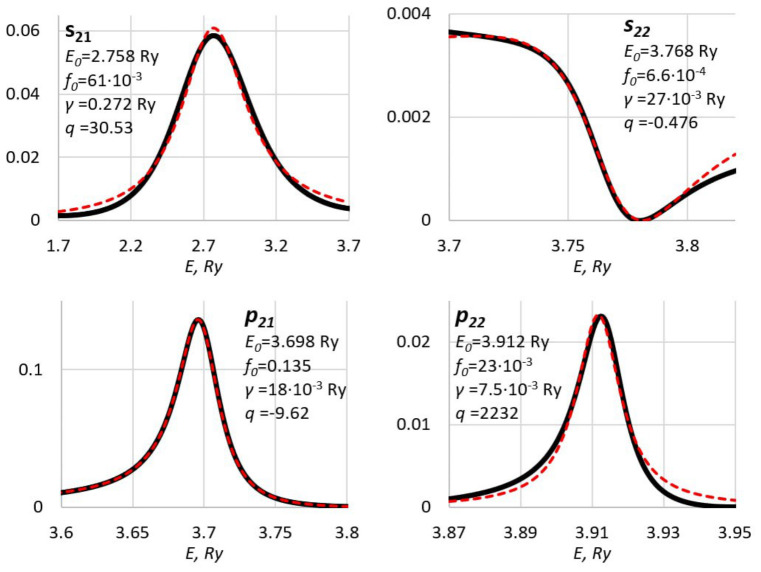
Examples of peak fitting using the Fano equation for the oscillator strengths of transitions from the ground impurity state $s_{11}$ to the resonant impurity states $s_{21}$, $s_{22}$, $p_{21}$, and $p_{22}$. L=30 nm, z=0.05 L. Black solid lines correspond to calculated curves, red dash lines corespond to approximated curves.

One can see that most of the peaks have a shape close to Lorentzian (though sometimes showing visible asymmetry), with the only exception being the transition to the $s_{22}$ state, which looks more like a well than a peak. In terms of the Fano resonance formula, this means that the transition to the $s_{22}$ state is highly resonant (low |q|), while other transitions are lowly resonant (high |q|). This allowed us to approximate the vicinity of specific curves around the transition energies using Equation (10) and derive key parameters for each peak. Figure 3 demonstrates the calculated curve fragments superimposed on the approximated curves.

As already mentioned, the characteristics of these curves are as follows. (1) γ from Equation (10) is denoted as broadening. In the case of a perfectly Lorentzian shape (q→±∞), this is the half-width at half-height of the peak. (2) $f_{0}$ is the “peak maximum”—for the Lorentzian shape, this is literally the maximum peak magnitude, whereas for smaller values of the Fano parameter *q*, the difference between $f_{0}$ and the maximum peak magnitude can be very significant. Also, for an ideal Lorentzian, the complete integral magnitude of the peak is equal to the product of $f_{0}$ and γ, and can be interpreted as an equivalent of complete absorption that corresponds to a specific transition for all energies.

**Figure 4:**
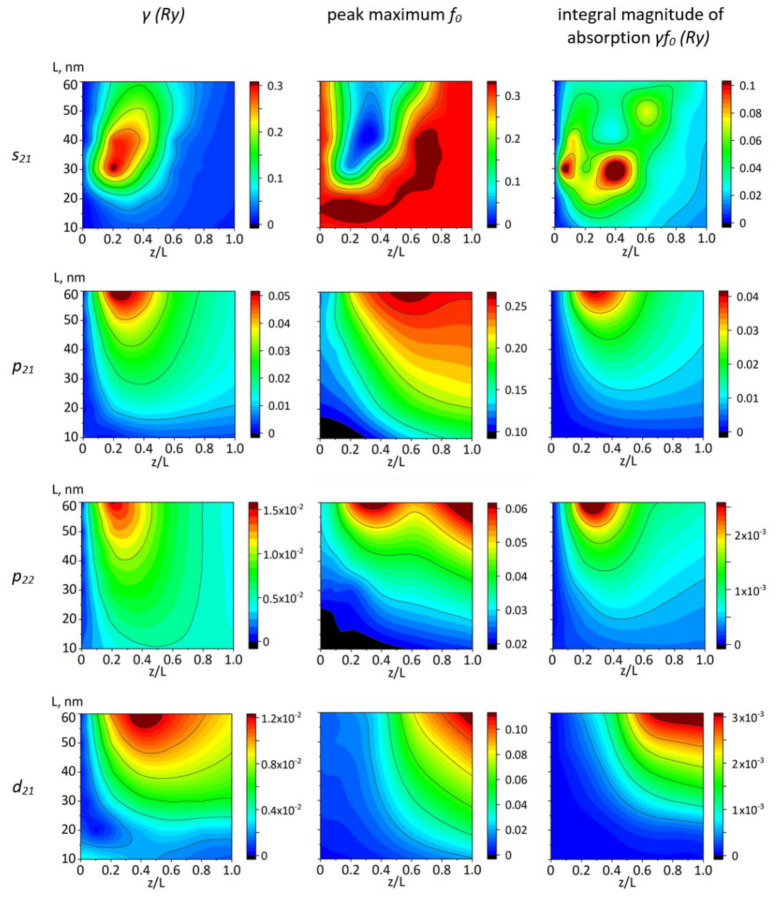
Peak characteristics of the intra-center absorption from the ground impurity state $s_{11}$ to four resonant states: first line—$s_{21}$, second line—$p_{21}$, third line—$p_{22}$, fourth line—$d_{21}$. Characteristics: first column—peak broadening γ (Ry), second column—peak maximum $f_{0}$ (unitless for oscillator strength) and third column—integral magnitude of absorption $\gamma f_{0}$ (Ry).

In Figure 4, we show the characteristics of the transition peaks from the ground impurity state $s_{11}$ to four resonant states ($s_{21}$, $p_{21}$, $p_{22}$, and $d_{21}$) for well widths from *L* = 10 nm to 60 nm and for the impurity position z/L from 0 to 1 (see Figure 1). It consists of temperature plots; each presents the dependence of a specific characteristic for a specific transition on two parameters. The numerical error for the presented parameters was not calculated; however, the fitting accuracy can be estimated visually in Figure 3 by comparing the fitted and calculated curves. We certify that the fitting accuracy is, in most cases, better than that of the presented graphs. Results for the transition to the $s_{22}$ state are not presented, as its shape is far from Lorentzian and cannot be interpreted visually. We do not present numerical results for the Fano parameters; instead, we analyze them qualitatively, as calculating them with the described model appears to introduce excessive numerical error.

### 6.1. Fano Parameter

Graphically, the Fano parameter *q*, as defined in the original work [18], quantifies the asymmetry of the peak profile. For an ideally symmetric peak, its absolute value approaches infinity, often resulting in extremely large magnitudes during numerical fitting. Consequently, minor variations in an almost-symmetric peak shape can trigger substantial absolute and relative fluctuations (see the various panels of Figure 3), posing significant challenges for both its numerical and graphical representation. On the other hand, our analysis of its behavior yields qualitative conclusions that do not rely on exact numerical evaluation. Therefore, to avoid unnecessary confusion, we have chosen not to provide a direct graphical representation of the parameter *q*. All the situations considered give us a qualitatively similar picture: the peaks $s_{21}$, $p_{21}$, $p_{22}$, and $d_{21}$ are mostly Lorentzian in shape, with a sufficiently large value of |q|, which implies low interference with some other kind of scattering.

The only state with high interference is $s_{22}$, which does not even produce a peak but a well in the spectrum. The visual analysis of spectra indicates that, within our model, the Fano parameter of the peaks reflects interference between neighboring peaks with the same first quantum number, as *s*-peaks interfere with other *s*-peaks, *p*-peaks with other *p*-peaks, etc. As the $s_{11}$–$s_{21}$ transition peak is the broadest and largest in integral magnitude, it strongly interferes with the $s_{11}$–$s_{22}$ transition as a background process and turns its peak into a well. *p*-peaks are relatively narrow, and their interference results only in slight asymmetry. The interaction of impurity WFs with the sub-band apparently results in peak broadening and does not directly influence the Fano parameter in our mathematical model.

### 6.2. Peak Maximum

Generally, wider quantum wells produce higher peaks. An exception is the transition to the first resonant state $s_{21}$, which has a kind of peak minimum at *L* = 30 nm, *z* = 0.3 L, that is, for an impurity located approximately in the middle between the QW center and the barrier. This can be explained by our results in [21] (see Figure 6 in the reference and the discussion below). The WF of the ground impurity state $s_{11}$ shifts to the right following the impurity center, while the WF of the first resonant state moves to the left, causing them to separate in space. As a result, the overlap integral between the wave functions of states $s_{11}$ and $s_{21}$ in the matrix element of Equation (3) decreases. This is most clearly observed for the impurity atom located exactly in the middle between the QW center and the barrier. It is noteworthy that the lowest peaks in this area are also the broadest. A spectrum in Figure 2 reflects exactly this situation (with a low but broad $s_{21}$ peak); in most other situations, the $s_{21}$ peak is also the largest, stably reaching values close to the theoretical maximum for the oscillator strength of 1/3 (as the transitions are prohibited in 2 of 3 spatial dimensions). For states located above $s_{21}$, the effect of different displacements of wave functions for the initial and final states also exists, but is expressed much less strongly.

### 6.3. Peak Broadening

For all states and QW widths, the peak broadening exhibits a consistent dependency on the impurity position: it is minimized when the impurity is at the well center, reaches a maximum when the impurity is located between the center and the barrier, and subsequently decreases monotonically as the impurity moves closer to the barrier interface. This trend qualitatively agrees with the results presented for Si/SiGe quantum wells in [24] (see Figure 1 therein), where the broadening is attributed to the hybridization, or coupling, between the impurity states and the lower sub-band states.

Following this line of reasoning, when the impurity is located precisely at the well center, the corresponding WFs have opposite parities with respect to the inversion center, resulting in a vanishing spatial overlap that prevents hybridization. In this symmetric configuration, the resonant impurity WF behaves nearly identically to a localized state. However, when the impurity is displaced from the center, the structural symmetry is broken; the impurity state mixes with the sub-band continuum, which induces energy broadening. As the impurity moves even further toward the barrier, its binding energy decreases (consistent with our previous findings in [21]), the energy separation between the impurity state and the lower sub-band edge increases, and the coupling strength subsequently diminishes. Most of the transitions have broader peaks for wider wells for the obvious reason that the energy distance between the sub-bands and, accordingly, between the impurity and lower sub-band state is less for wider wells, so the coupling is greater for the wider well. An exception is for the first resonant state $s_{21}$, which has the broadest peak at *L* = 30 nm.

As noted in the ’Assumptions and Limitations’ section, the actual peak broadening observed in experimental spectra includes not only the resonant broadening investigated in this work, but also linewidth contributions from other mechanisms such as phonon scattering, interface roughness, impurity distribution inhomogeneity, and finite-temperature effects. Assuming independent stochastic processes with Lorentzian profiles, the total line shape is expected to remain Lorentzian, with a total linewidth equal to the direct sum of the broadenings from each individual mechanism [25].

Experimental studies on similar structures [26,27] report a peak broadening of approximately 1 meV for intra-center absorption between non-resonant states. Consequently, for transitions to resonant states, we expect the experimental linewidths to be no less than this value. In particular, the ultra-narrow peaks predicted for center-doped structures are expected to exhibit a width of at least 1 meV in practice. The actual corrections, however, will ultimately depend on the growth technology and structural quality of the fabricated nanostructures. Furthermore, at elevated temperatures, electrons will be thermally distributed among multiple impurity states and quantum-well sub-bands. While this population redistribution does not directly alter oscillator strengths, it complicates the absorption spectrum.

The behavior of the integral magnitude of the peak $f_{0}\gamma$ in most cases is trivial and follows the behavior of $f_{0}$ and γ, with the interesting exception of $s_{21}$, where the maximum of γ approximately corresponds to the minimum of $f_{0}$. Thus, two local maxima are formed at *L* = 30 nm: one closer to the center of the well and another closer to the barrier, but still within the well.

## 7. Conclusions

The interaction between the resonant impurity states in the quantum well and the sub-band below them broadens the absorption peaks for intra-center transitions. The peaks are wider when they are closer to the edge of the lower sub-band. Our calculations for GaAs/AlGaAs wells show broadening of up to 0.3 Ry, with 1 Ry = 5.8 meV for our material. The shape of the peaks is mostly Lorentzian, with asymmetry caused by the interference with the neighboring peaks that have the same azimuthal quantum number “a”. In the case of very wide first peaks, the next impurity state with the same energy can produce a well instead of a peak that can be described in terms of Fano resonance as a result of interference of scatterings to neighboring excited impurity states. Wider broadening, as well as a higher peak maximum for most states, is predicted for the wider wells (which also correspond to lower energies). The exception is the first resonant state, which exhibits atypical behavior, with maximum broadening for a well width of about 30 nm, when doped halfway between the well center and the barrier. For the well doped exactly to the well center, the broadening is very small because of the symmetry but grows fast when an impurity atom is displaced from the center towards a barrier.

The results could be interesting for engineers designing integrated photonic devices that use single-photon generation, for applications of Fano resonance, and for the fundamental study of the interaction between confined quantum states and the continuous spectrum.

## Data Availability

The original contributions presented in this study are included in the article. Further inquiries can be directed to the corresponding author.

## Abbreviations

The following abbreviations are used in this manuscript:

QW	Quantum Well
WF	Wave Function
